# Low-mass-ion discriminant equation (LOME) for ovarian cancer screening

**DOI:** 10.1186/s13040-016-0111-7

**Published:** 2016-10-12

**Authors:** Jun Hwa Lee, Byong Chul Yoo, Yun Hwan Kim, Sun-A Ahn, Seung-Gu Yeo, Jae Youl Cho, Kyung-Hee Kim, Seung Cheol Kim

**Affiliations:** 1 Division of Gynecologic Oncology, Department of Obstetrics and Gynecology, Ewha Womans University Mokdong Hospital, College of Medicine, Ewha Womans University, Seoul, Republic of Korea; 2 Colorectal Cancer Branch, Research Institute, National Cancer Center, Goyang, Gyeonggi Republic of Korea; 3 Department of Radiation Oncology, Soonchunhyang University College of Medicine, Cheonan, Republic of Korea; 4 Department of Genetic Engineering, Sungkyunkwan University, Suwon, Gyeonggi Republic of Korea; 5 Omics Core Laboratory, Research Institute, National Cancer Center, Goyang, Gyeonggi Republic of Korea

**Keywords:** Ovarian cancer, Screening, Serum profiling, MALDI-TOF mass spectrometry, Pattern recognition

## Abstract

**Background:**

A low-mass-ion discriminant equation (LOME) was constructed to investigate whether systematic low-mass-ion (LMI) profiling could be applied to ovarian cancer (OVC) screening.

**Results:**

Matrix-assisted laser desorption/ionization-time of flight (MALDI-TOF) mass spectrometry was performed to obtain mass spectral data on metabolites detected as LMIs up to a mass-to-charge ratio (*m/z*) of 2500 for 1184 serum samples collected from healthy individuals and patients with OVC, other types of cancer, or several types of benign tumor. Principal component analysis-based discriminant analysis and two search algorithms were employed to identify discriminative low-mass ions for distinguishing OVC from non-OVC cases. OVC LOME with 13 discriminative LMIs produced excellent classification results in a validation set (sensitivity, 93.10 %; specificity, 100.0 %). Among 13 LMIs showing differential mass intensities in OVC, 3 metabolic compounds were identified and semi-quantitated. The relative amount of LPC 16:0 was somewhat decreased in OVC, but not significantly so. In contrast, _D,L_ **-**glutamine and fibrinogen alpha chain fragment were significantly increased in OVC compared to the control group (*p* = 0.001 and 0.002, respectively).

**Conclusion:**

The present study suggested that OVC LOME might be a useful non-invasive tool with high sensitivity and specificity for OVC screening. The LOME approach could enable screening for multiple diseases, including various types of cancer, based on a single blood sample. Furthermore, the serum levels of three metabolic compounds—_D,L_ **-**glutamine, LPC 16:0 and fibrinogen alpha chain fragment—might facilitate screening for OVC.

**Electronic supplementary material:**

The online version of this article (doi:10.1186/s13040-016-0111-7) contains supplementary material, which is available to authorized users.

## Background

The 5-year survival rate of ovarian cancer is high (~90 %) if detected in the early stages, but this rate drops sharply to nearly 30 % with diagnosis at an advanced stage. The problem remains that more than two thirds of ovarian cancer patients present with advanced-stage disease [1]. Effective screening methods to facilitate early detection of ovarian cancer at a curable stage would reduce the mortality rate of this disease.

Previous studies have shown that screening for serum cancer antigen 125 (CA-125), transvaginal ultrasonography, or a combined strategy have failed to reduce the risk of diagnosis at an advanced stage or to improve the survival of female patients participating in clinical trials [2]. Due to the location of the ovaries, invasive surgery and removal of the ovaries are necessary for definitive diagnosis of ovarian cancer. Therefore, high specificity is mandatory in screening tests because false positivity can cause unnecessary operations and surgical complications. Furthermore, the low incidence of ovarian cancer makes it essential for screening tests to have a high degree of specificity [3]. At present, there are no screening methods that are accredited and recommended by a professional society for ovarian cancer in the general population [1]. Identification of biomarkers with high sensitivity and higher specificity would facilitate development of effective screening methods for ovarian cancer.

We analyzed low-mass ions (LMIs) in serum, which can provide information regarding metabolic disturbance, using matrix-assisted laser desorption/ionization-time of flight (MALDI-TOF) mass spectrometry. The metabolome is essentially an accumulation of all metabolites and the final products of cellular processes [4]. Understanding metabolic changes in body fluids is important for detecting and monitoring disease [5]. Based on the LMI profiles, we developed the LOw-Mass-ion discriminant Equation (LOME) as a novel method for ovarian cancer screening. Here, we describe use of the LOME for detection of ovarian cancer.

## Methods

### Study population

A total of 1,184 serum samples (Table 1, Additional file 1) were collected from healthy female control subjects (controls) and female patients with ovarian cancer (OVC), colorectal cancer (CRC), gastric cancer (GC), benign uterine tumor (BUT), benign ovarian tumor (BOT), precancerous cervical lesion (PCL), breast cancer (BRC), benign breast tumor (BBT), uterine cervical cancer (UCC), or endometrial cancer (EMC). Serum was collected before surgery or chemotherapy to prevent any effects of anesthetic or anticancer agents on serum low-mass ions (LMIs). UCC and EMC cases were not included in the training process, because the numbers of cases were relatively small. Table 2 shows the locations of sample collection and the number of samples collected at each site. Informed consent was obtained from all healthy individuals and patients, and the institutional review board of each participating institution approved the research protocol. The part of research source was provided by Korea gynecologic cancer bank through Bio & Medical Technology Development program of the MSIP, Korea.

**Table 1:** Number, disease stage, and age information of the study population

	Number	Stage					Age (years)	
	Total	0	I	II	III	IV	Mean ± SD	Range
Control	276						50.3 ± 10.7	19 – 86
OVC	89		18	9	51	11	55.5 ± 10.3	27 – 77
CRC	237	2	67	70	87	11	63.4 ± 11.8	30 – 87
GC	139	2	96	11	15	15	59.1 ± 13.8	28 – 86
BUT	83						45.8 ± 8.2	25 – 70
BOT	71						42.0 ± 13.4	20 – 83
PCL	88						42.1 ± 11.9	22 – 79
BRC	93	11	40	34	8		47.8 ± 9.2	29 – 69
BBT	65						45.4 ± 10.0	19 – 62
UCC	33		20	10	1	2	50.7 ± 14.8	26 – 82
EMC	10		8		2		53.6 ± 7.9	40 – 66

More detailed information for individual samples was tabulated in Additional file 1

*SD* standard deviation, *OVC* ovarian cancer, *CRC* colorectal cancer, *GC* gastric cancer, *BUT* benign uterine tumor, *BOT* benign ovarian tumor, *PCL* precancerous cervical lesion, *BRC* breast cancer, *BBT* benign breast tumor, *UCC* uterine cervical cancer and *EMC* endometrial cancer

**Table 2:** Institutions from which samples were collected

	Number	Institution (Address)
Control	193	National Cancer Center Hospital (323 Ilsan-ro, Ilsandong-gu, Goyang-si Gyeonggi-do, 410–769, Korea)
	83	Dong-A University Medical Center (26 Daesingongwon-ro, Seo-gu, Busan, 602–715, Korea)
OVC	8	Seoul National University Hospital (101 Daehak-ro, Jongno-gu, Seoul, 110–744, Korea)
	22	Samsung Medical Center (81 Irwon-ro, Gangnam-gu, Seoul, 135–710, Korea)
	31	National Cancer Center Hospital
	28	Ewha Woman’s University Mokdong Hospital (1071, Anyangcheon-ro, Yangcheon-gu, Seoul, 158–710, Korea)
CRC	74	National Cancer Center Hospital
	32	Daehang Hospital (2151 Nambusunhwan-ro, Seocho-gu, Seoul, 137–820, Korea)
	131	Dong-A University Medical Center
GC	139	Dong-A University Medical Center
BUT	83	Ewha Woman’s University Mokdong Hospital
BOT	71	Ewha Woman’s University Mokdong Hospital
PCL	88	Ewha Woman’s University Mokdong Hospital
BRC	93	National Cancer Center Hospital
BBT	65	National Cancer Center Hospital
UCC	33	Ewha Woman’s University Mokdong Hospital
EMC	10	Ewha Woman’s University Mokdong Hospital

*OVC* ovarian cancer, *CRC* colorectal cancer, *GC* gastric cancer, *BUT* benign uterine tumor, *BOT* benign ovarian tumor, *PCL* precancerous cervical lesion, *BRC* breast cancer, *BBT* benign breast tumor, *UCC* uterine cervical cancer and *EMC* endometrial cancer

### Construction of a LOME for OVC screening

The procedures for constructing a LOME for OVC screening were similar to those described in our previous report [6]. They are briefly repeated here, with an emphasis on major changes.

#### MALDI-TOF sample preparation & analysis

MALDI-TOF (Autoflex Speed, Buker Daltonik GmbH, Bremen, Germany) analysis was performed as described previously [6]. Serum samples (25 μL) were extracted using 100 μL of methanol/chloroform mixture (2:1, v/v) for 10 min at room temperature after vigorous vortexing. The mixture was centrifuged at 6000  ×  *g* for 10 min at 4 °C. The supernatant was dried completely in a concentrator for 1 h and resolved in 30 μL of 50 % acetonitrile/0.1 % trifluoroacetic acid (TFA) on a vortex mixer for 30 min. The methanol/chloroform extract was mixed (1:12, v/v) with an α-cyano-4-hydroxycinnamic acid solution in 50 % acetonitrile/0.1 % TFA, and 1 μL of the mixture was spotted on the MALDI target for analysis. For fixed focus mass and laser intensity, each sample was analyzed six times using different extractions and data acquisition times.

#### Two-stage training scheme

Serum samples were approximately trisected into Sets A_1_, A_2_, and B (Table 3). The samples of each clinical stage were divided almost evenly into these three sets. Sets A (A_1_∪A_2_) and B are the training and validation sets, respectively. The weighting factors for individual LMIs were calculated based on Set A_1_ only. The training set was then expanded to reduce overfitting by incorporating Set A_2_, which was independent of Set A_1_. The discriminative LMIs were determined based on Set A.

**Table 3:** Sample sets

	Total	Training set		Validation set
		Set A1	Set A2	Set B
Control	276	92	92	92
OVC	89	30	30	29
CRC	237	79	79	79
GC	139	47	46	46
BUT	83	27	28	28
BOT	71	23	24	24
PCL	88	30	29	29
BRC	93	31	31	31
BBT	65	22	21	22
UCC	33			33
EMC	10			10

*OVC* ovarian cancer, *CRC* colorectal cancer, *GC* gastric cancer, *BUT* benign uterine tumor, *BOT* benign ovarian tumor, *PCL* precancerous cervical lesion, *BRC* breast cancer, *BBT* benign breast tumor, *UCC* uterine cervical cancer and *EMC* endometrial cancer

#### Weighting factors for individual LMIs

MALDI-TOF measurements were carried out six times on each sample. Principal component analysis-based discriminant analysis (PCA-DA) was performed to separate the OVC group from the Non-OVC group in Set A_1_ using the MarkerView software (AB SCIEX, Foster City, CA). The six measurements of Set A_1_ were analyzed individually, and one measurement with the highest separation performance was assigned as the reference mass spectrum. PCA-DA on the reference mass spectrum yielded a weighting factor vector termed a loading vector.

#### Data preprocessing

Importing mass spectra into the MarkerView software produces a peak table, which consists of one mass-to-charge ratio (*m/z*) column and one intensity column per sample. To obtain the discriminant score (DS) of a sample by assigning the weighting factors derived from the reference mass spectrum, the mass spectrum of the sample should be aligned with the reference mass spectrum, i.e., the *m*/*z* column of the former should be identical to that of the latter. The preprocessing steps were as follows: 1) The mass spectra of all samples (five measurements per sample) were aligned with the reference mass spectrum by importing each mass spectrum together with the reference mass spectrum into the MarkerView software (import settings: mass tolerance, 300 ppm; minimum required response, 10.0; and maximum number of peaks, 10000). But the resulting peak table was not completely aligned: that is, the *m/z* column of the reference mass spectrum plus a mass spectrum was not identical to that of the reference mass spectrum only. 2) The aligned mass spectra were realigned with the reference mass spectrum with a mass tolerance of 300 ppm. 3) The realigned mass spectra were normalized using the “Normalization Using Total Area Sums” scheme (See MarkerView Software Reference Manual for details). 4) The normalized mass spectra were Pareto-scaled. 5) The Pareto-scaled mass spectra were multiplied by the weighting factors. 6) The five weighted mass spectra obtained per sample were averaged.

#### Preliminary LMI candidates

PCA-DA DS was calculated as the weighted sum of the Pareto-scaled intensities of all LMIs (≤ 10000 LMIs). However, most LMIs made trivial contributions to the DS. Search algorithm 1 revealed the *P* preliminary LMI candidates with the following two criteria: 1) LMIs with weighted intensities that have a magnitude >  0.1 for each intensity column in the weighted reference mass spectrum. 2) LMIs selected simultaneously in more than half of the intensity columns in the reference mass spectrum.

#### Discriminative LMIs

**Fig. 1:**
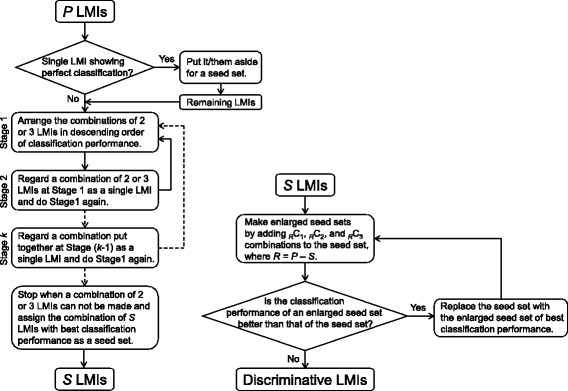
Search algorithm 2

The discriminative LMIs were searched based on the averaged mass spectra of Set A and the *P* preliminary LMI candidates. Search algorithm 2 (Fig. 1) consisted of the following steps. 1) Whether there was a single LMI with a sensitivity and specificity of 100 % for Set A was determined. 2) The sums of the sensitivity and specificity for _*P*_C_2_ and _*P*_C_3_ combinations were calculated. 3) The combination of two or three LMIs with the maximum sum of sensitivity and specificity was put aside and Step 2) was iterated with the remaining LMIs until one or no LMI remained. 4) A combination of two or three LMIs was considered a single LMI and Steps 2) – 3) were iterated. 5) Step 4) was iterated. The combination put together at the preceding iteration was considered a single LMI at the subsequent iteration. 6) The combination of *S* LMIs with the maximum sum of sensitivity and specificity was assigned as a seed set. 7) The _*R*_C_1_, _*R*_C_2_, and _*R*_C_3_ combinations were added to the seed set, where *R*  =  *P* – *S*. 8) The enlarged seed set with the maximum sum of sensitivity and specificity was assigned as a new seed set if the enlarged seed set was better than the former seed set in terms of the sum of sensitivity and specificity, and Step 7) was iterated with the remaining LMIs. 9) The last updated seed set was assigned as the discriminative LMIs. The LOME with discriminative LMIs can be expressed as follows:

$$ \mathrm{D}\mathrm{S}={\displaystyle {\sum}_{\mathrm{disciminative}\ \mathrm{LMIs}}\Big(\mathrm{Pareto}-\mathrm{scaled}\kern0.5em \mathrm{intensity}}\times \mathrm{Weighting}\kern0.5em \mathrm{factor}\Big) $$

When the number of combinations with the maximum sum of sensitivity and specificity was >  1, one was selected using the following two criteria: Priority 1) When the numbers of LMIs in the combinations showing the same sum of sensitivity and specificity were different, the combination with the fewest LMIs was selected. This choice resulted in a better performance in this study. Priority 2) When the numbers of LMIs in the combinations were equal, the combination with the largest Fisher’s discriminant ratio was selected.

### Validation of LOME for OVC screening

Set B was reserved for the validation process. Sets A and B were mutually exclusive. The mean DSs for Set B were calculated based on the averaged mass spectra of Set B and the discriminative LMIs derived from Set A. The mean DS of a sample was the sum of the averaged intensities of the discriminative LMIs. A decision was made based on the sign of the mean DS, i.e., plus/minus DS indicated screen-positive/negative, respectively.

### Identification of LMIs

The methanol/chloroform extract was dried, and then reconstituted in 0.1 % formic acid (FA) and subjected to liquid chromatography - mass spectrometry (LC-MS) analysis, using Eksigent ultraLC 110-XL system coupled to an AB Sciex Triple TOF 5600+ system, equipped at the front end with a DuoSpray ion source. For the ultraLC separation, the sample was loaded into an Atlantis T3 sentry guard cartridge (3 μm, 2.1 × 10 mm; Waters), and then separation was performed in an Atlantis T3 column (3 μm, 2.1 × 100 mm; Waters) in a two-step linear gradient (solvent A, 0.1 % FA in water; solvent B, 100 % Acetonitrile; with 1 % solvent B for 2 min, 1 to 30 % B for 6 min, 30 to 90 % B for 8 min, 90 % B for 4 min, 90 to 1 % B for 1 min and 9 min in 1 % B). The MS system was set to perform one full scan (50 to 1,200 m/z range) followed by tandem mass spectrometry (MS/MS) of the 10 most-abundant parent ions (mass tolerance, 50 mDa; collision energy, 35 %). The MS and MS/MS spectra were submitted to the Formula Finder computational tools (Sciex) that proposes probable elemental compositions within a specified mass tolerance of a given mass-to-charge ratio using the PeakView software (Sciex). Using metabolite databases comprising Human Metabolome Database (HMDB), specific compounds were found for the given m/z, listed in rank order based on the MS and MS/MS data. A proteomic MS/MS analysis was performed using the ProteinPilot software (Sciex).

### Statistical analysis

Between-group differences were analyzed using the non-parametric Mann–Whitney *U*-test, and significance was set at *P* < 0.05.

## Results

### Preliminary LMI candidates

**Fig. 2:**
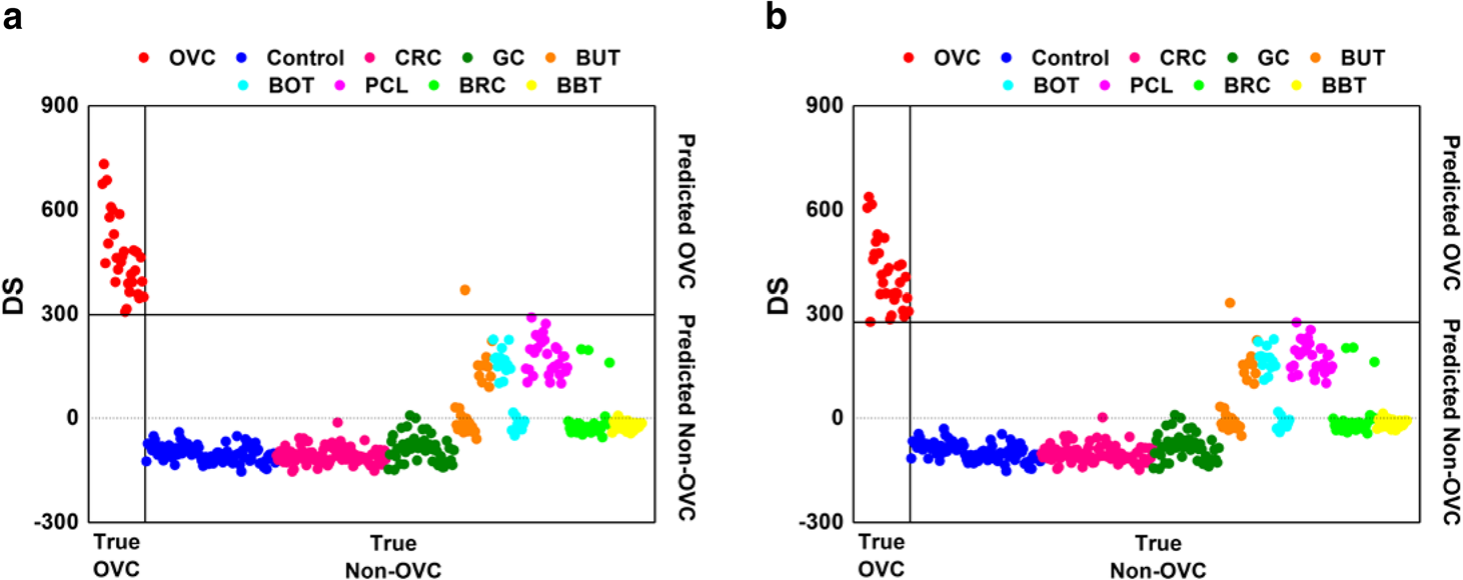
Classification results for the reference mass spectrum. **a** Principal component analysis-based discriminant analysis. **b** Preliminary low-mass-ion candidates. DS, discriminant score; OVC, ovarian cancer; CRC, colorectal cancer; GC, gastric cancer; BUT, benign uterine tumor; BOT, benign ovarian tumor; PCL, precancerous cervical lesion; BRC, breast cancer; and BBT, benign breast tumor

The results of classification for the reference mass spectrum using PCA-DA and the preliminary LMI candidates are shown in Fig. 2a and b, respectively. Excellent separation performance was observed with the threshold DS of the solid horizontal line. A total of 10000 LMIs were involved in the PCA-DA DSs. Search algorithm 1 selected 176 preliminary LMI candidates. Although only 1.76 % of LMIs were used to compute the DSs, the separation capability remained unchanged. Further, comparison of Fig. 2a and b indicated that the marked reduction in number of LMIs did not lead to marked variation in the DS range.

### Discriminative LMIs

**Fig. 3:**
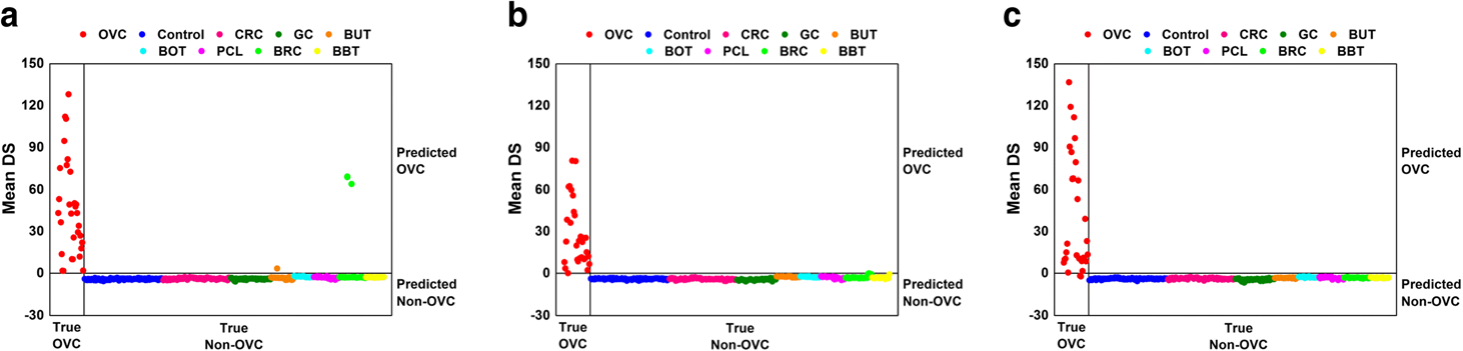
Classification results using the 13 discriminative low-mass ions. **a** Set A_1_. **b** Set A_2_. **c** Set B. Sets A_1_ and A_2_ are the training sets for the two-stage training scheme. Set B is the validation set. DS, discriminant score; OVC, ovarian cancer; CRC, colorectal cancer; GC, gastric cancer; BUT, benign uterine tumor; BOT, benign ovarian tumor; PCL, precancerous cervical lesion; BRC, breast cancer; and BBT, benign breast tumor

Search algorithm 2 yielded 13 discriminative LMIs for separating OVC from Non-OVC (Table 4). The classification results for all samples using the discriminative LMIs are shown in Fig. 3, and Table 5 presents a summary of the classification performance. Sensitivity was 93.10 % and specificity was 100.0 % for Set B, whereas low specificities were observed for UCC and EMC cases that were not included in the training process.

**Table 4:** Discriminative LMIs for separating OVC from Non-OVC

Mass value in m/z						
21.1873	37.5142	38.6222	147.1669	175.1585	190.7493	709.7879
37.0311	37.7989	84.8716	171.4024	188.8544	230.0197

**Table 5:** Classification performance using the 13 discriminative LMIs

	Training set		Validation set
	Set A1	Set A2	Set B
Sensitivity (%)	100.0	100.0	93.10
Specificity (%)	99.15	100.0	100.0
PPV (%)	90.91	100.0	100.0
NPV (%)	100.0	100.0	99.43
Specificity of UCC (%)			48.48
Specificity of EMC (%)			50.00

*PPV* positive predictive value, *NPV* negative predictive value

### Addition of LPC 16:0, LPC18:0 and fibrinogen α-chain fragment

**Fig. 4:**
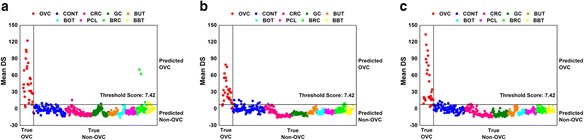
Classification results using the 13 discriminative and 3 identified low-mass ions. **a** Set A_1_. **b** Set A_2_. **c** Set B. Sets A_1_ and A_2_ are the training sets for the two-stage training scheme. Set B is the validation set. DS, discriminant score; OVC, ovarian cancer; CRC, colorectal cancer; GC, gastric cancer; BUT, benign uterine tumor; BOT, benign ovarian tumor; PCL, precancerous cervical lesion; BRC, breast cancer; and BBT, benign breast tumor

The effect of the three identified LMIs—lysophosphatidylcholine (LPC) 16:0 (496.5220 m/z), LPC 18:0 (524.5837 m/z), and fibrinogen α-chain fragment (1466.7073 m/z)—on the classification performance was investigated. A LOME incorporating only the three LMIs did not provide good classification performance (sensitivity, 41.38 %; specificity, 77.49 % for Set B; specificity of UCC, 72.73 %; specificity of EMC, 70.00 %). As a next step, a LOME augmented with the three LMIs was evaluated. Figure 4 presents the classification results using the 13 discriminative plus the 3 identified LMIs, and Table 6 shows the corresponding classification performance. A threshold score was trained based on Set A and all decisions were made on Set B with the trained threshold score. While the sensitivity and specificity for Set B worsened slightly, the specificities of UCC and EMC were greatly improved.

**Table 6:** Classification performance using the 13 discriminative and 3 identified LMIs

	Training set		Validation set
	Set A1	Set A2	Set B
Sensitivity (%)	90.00	90.00	89.66
Specificity (%)	97.15	96.57	98.01
PPV (%)	72.97	69.23	78.79
NPV (%)	99.13	99.12	99.14
Specificity of UCC (%)			90.91
Specificity of EMC (%)			100.0

*PPV* positive predictive value, *NPV* negative predictive value

### Identification and semi-quantification of LMIs

**Fig. 5:**
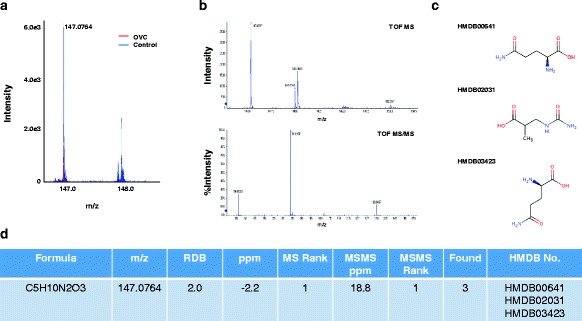
Selection of candidate metabolites for the LMI with 147.0764 m/z. **a** Mass spectra of 147.0764 m/z. MS and MS/MS pattern analyses were performed using a Triple-TOF mass spectrometer. The LMI with 147.1699 m/z in the MALDI mass spectrum was shifted to 147.0764 m/z in the Triple-TOF mass spectrum. The intensity of the LMI with 147.0764 m/z was significantly higher in the control group (peaks in blue) compared to the OVC group (peaks in red). **b** MS/MS analysis of the LMI with 147.0764 m/z. **c** Structure of HMDB00641 (_L_-glutamine), HMDB02031 (ureidoisobutyric acid) and HMDB03423 (_D_-glutamine). **d** HMDB number of the LMI with 147.0764 m/z. Based on the MS/MS analytic pattern, three metabolic compounds—HMDB00641 (_L_-glutamine), HMDB02031 (ureidoisobutyric acid) and HMDB03423 (_D_-glutamine)—for the LMI with 147.0764 m/z were selected

**Fig. 6:**
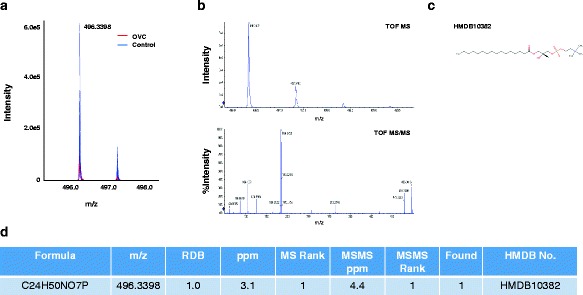
Identification of the LMI with 496.3398 m/z. **a** Mass spectra of 496.3398 m/z. MS and MS/MS pattern analyses were performed using a Triple-TOF mass spectrometer. The intensity of the LMI with 496.3398 m/z was significantly higher in the control group (peaks in blue) compared to the OVC group (peaks in red). **b** MS/MS analysis of the LMI with 496.3398 m/z. **c** Structure of HMDB10382 (LPC 16:0). **d** HMDB number of the LMI with 496.3398 m/z. Based on the MS/MS analytic pattern, the metabolic compound with 496.3398 m/z was identified as HMDB10382 (LPC 16:0)

**Fig. 7:**
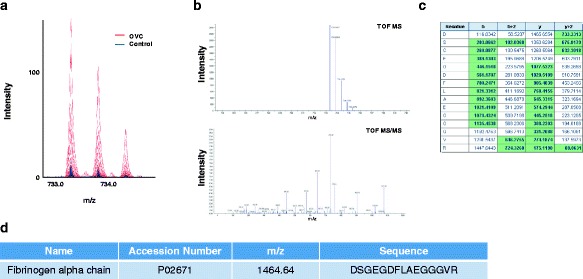
Identification of the LMI with 1464.64 m/z. **a** Mass spectra of 1464.64 m/z. MS and MS/MS pattern analyses were performed using a Triple-TOF mass spectrometer. The intensity of the LMI with 1464.64 m/z was significantly higher in the OVC group (peaks in red) compared to the control group (peaks in blue). **b** MS/MS analysis of the LMI with 1464.64 m/z. **c** Peptide sequence analysis demonstrating that the LMI with 1464.64 m/z was the fibrinogen alpha chain fragment. Protein identification based on the MS/MS analytic pattern. **d** The LMI with 1464.64 m/z was identified as the fibrinogen alpha chain fragment

To predict molecular formulas that match LMIs, the Formula Finder computational tools and ProteinPilot software (Sciex) were used. The resulting MS and MS/MS spectra were compared with compound details, and _D,L_-glutamine and LPC 16:0 were identified (Figs. 5 and 6). The LMI with 147.1699 m/z selected for composing OVC LOME was shifted to 147.0764 m/z on the Triple-TOF mass spectrum (Fig. 5a). Fibrinogen alpha chain fragment also predicted possible metabolites with accurate masses and isotopic patterns at 147.0764, 496.3398 and 1464.64 m/z (Fig. 7). Although LPC 16:0 and fibrinogen alpha chain fragment were not included in the OVC LOME, additional information on LPC 16:0 and fibrinogen alpha chain fragment increased its discrimination power (Table 6).

**Fig. 8:**
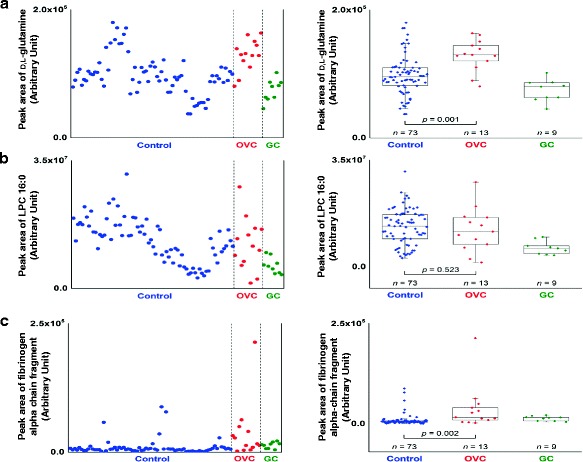
Semi-quantitative analysis of _D,L_-glutamine, LPC 16:0 and fibrinogen alpha chain fragment using peak areas in Triple-TOF mass spectra. Control (*n* = 73), OVC (*n* = 13), and GC (*n* = 9) samples were further analyzed using Triple-TOF MS for semi-quantitation of the three identified LMIs [**a**_D,l_-glutamine, **b** LPC 16:0, and **c** fibrinogen alpha chain fragment]. Left panels show the peak areas of _D,l_-glutamine, LPC 16:0, and fibrinogen alpha chain fragment from individuals. Right panels are the results of statistical analysis demonstrating differential levels of the three molecules in the OVC group

To obtain more information on the relative levels of the three identified LMIs in OVC, control (*n* = 73), OVC (*n* = 13), and GC (*n* = 9) samples were further analyzed using Triple-TOF MS. Peak areas responsible for the three identified LMIs—_D,l_-glutamine (Fig. 8a), LPC 16:0 (Fig. 8b), and fibrinogen alpha chain fragment (Fig. 8c)—were calculated in individual samples. The mass peak areas of _D,L_-glutamine and fibrinogen alpha chain fragment were significantly increased in the OVC group compared to the control group (*p* = 0.001 and *p* = 0.002, respectively) (Fig. 8a, c). The mass peak area for LPC 16:0 was smaller in the OVC groups, albeit not significantly so (*p* = 0.523) (Fig. 8b). However, the LPC 16:0 level facilitated separation of OVC from other types of cancer, such as GC (Fig. 8b, right panel).

## Discussion

Metabolomics is the global assessment of endogenous small molecule metabolites within a biological system, and altered metabolism is well established as a hallmark of cancer, which contributes to tumorigenicity and malignancy [6, 7]. Many studies have shown increased rates of glycolysis, glutaminolysis, and lipid synthesis in cancers, suggesting that altered metabolism promotes tumor growth [8]. Metabolomics has been utilized to identify novel biomarkers that could be used to distinguish cancer patients from their counterparts without neoplasms [6, 7, 9–11]. Exploring metabolic signatures of biological specimens would aid in the early diagnosis of ovarian cancer and also clarification of disease pathogenesis. The advantages of this technology in the search for ovarian cancer screening methods also include the ability to identify numerous new potential biomarkers present at low concentrations in serum.

Search algorithm 2 for discriminative LMIs was newly devised in the present study. The previous algorithm [6] employed the sensitivity and specificity of each LMI, i.e., each LMI was sorted in descending order of the sum of sensitivity and specificity and then examined in that order in the search process. However, all decisions were made using the sensitivity and specificity of a combination of LMIs, rather than each LMI, in the present study. This novel algorithm checked many more combinations of LMIs than in our previous work before determining the discriminative LMIs.

Using MS/MS pattern analysis and calculation of mass peak area we identified and semi-quantitated three metabolic compounds in OVC (Figs. 5, 6, 7, 8). The mass peak area of LPC 16:0 was significantly decreased in the OVC group (Fig. 8b), whereas the relative amounts of D,L-glutamine and fibrinogen alpha chain fragment were significantly higher in the OVC group compared to the control group (Fig. 8a, c). Unfortunately, we were not able to determine the amounts of three metabolites in all samples listed in Table 3 because of the limited amount of individual samples. Therefore, further study needs to clarify an effect of small sample number of OVC. Glutamine is one of the major amino acids used by tumor cells for biosynthesis. Targeted inhibition of glutamine metabolism in cancers such as OVC and BRC has anti-tumorigenic effects [12–17]. Addition of glutamine to culture medium increases the proliferation rate of OVC cell lines [12, 13], whereas its absence induces reactive oxygen species and expression of endoplasmic reticulum stress proteins [12]. In the present study, we identified LMI with 147.0764 m/z as D,L-glutamine, and the mass peak area of D,L-glutamine was lower in OVC (Fig. 8). Our result suggests that the glutamine concentration in blood may be a useful index for screening OVC.

Recent studies have suggested that lysophospholipids bind to activate G protein-coupled receptors to initiate growth, proliferation, and survival pathways in cancer cells [18]. If lysophospholipids were released to the bloodstream, they might serve as cancer screening markers. Among lysophospholipids, LPC 16:0 has been reported as a potential biomarker not only for OVC but also for other types of cancer [19], and our previous and present studies confirmed its potential screening power for OVC [9] (Figs. 6 and 8). Although the relative concentration of LPC 16:0 in OVC was not significantly different from that in the control group, it enabled separation of OVC from other types of cancer, such as GC (Fig. 8b, right panel). However, the molecular mechanism(s) linked to downregulation of LPC 16:0 in OVC blood samples remain to be elucidated. Our recent MALDI-TOF analysis revealed that increased fibrinogen alpha chain fragment in blood was an important factor for screening for CRC [6]. In the present study, upregulation of fibrinogen alpha chain fragment was also found in blood from OVC patients (Fig. 8). Fibrinogen alpha chain fragment is considered an important regulator of inflammation [20]. Therefore, an increased level of fragmented fibrinogen alpha chain fragment in blood may be common to many types of cancer that are accompanied by inflammation [6, 21–23].

Despite the screening power of the OVC LOME, three points should be considered in further studies. First, the number of OVC samples was relatively small in this study. To validate and refine the current procedures and results, a larger set of serum samples is being collected from multiple centers in the Republic of Korea, and will be tested in a future study. Second, a decision of “indeterminate” may be introduced for subjects with a DS near the threshold score, so that an appropriate recommendation can be made. We expect also that the linkage between accumulated clinical data and LMI information will reduce the rate of indeterminate cases. Search algorithm 2 consisted of the germination (Steps 1–6) and growth (Steps 7–9) modules. It will be revised again to yield a more compact set of discriminative LMIs by including a shrinkage module. It was not of primary concern to compare several classifiers until now. But it would be one of future works. Third, fibrinogen alpha chain fragment is an important metabolite to discriminate disease group accompanied by inflammation. But it also shows very variable range depending on cancer type (e.g. it is higher in biliary tract cancer compared to CRC, lung cancer and inflammatory bowel disease, unpublished data), cancer stage [6] and so on. Therefore, fibrinogen alpha chain fragment might have a different weighting factor in construction of LOME depending on a type of disease, or might be ignored because of other strong discriminative metabolic factors.

## Conclusions

In conclusion, we developed a cancer-screening tool by profiling LMIs in the blood and applied it to CRC, BRC, and GC in our previous work [6]. This method showed high sensitivity and specificity, and could be applicable for OVC screening. Three metabolic compounds—D,L-glutamine, LPC 16:0 and fibrinogen alpha chain fragment—might be included in a metabolic index to screen for OVC, but three main points considered in this study should be clarified in further studies.

## Additional file

Additional file 1: More detailed information for individual samples. **Table S1.**Healthy Control Individuals Providing Sera for LMI Profiling. **Table S2.** Patients with OVC Providing Sera for LMI Profiling. **Table S3.** Patients with CRC Providing Sera for LMI Profiling. **Table S4.** Patients with GC Providing Sera for LMI Profiling. **Table S5.** Patients with BUT Providing Sera for LMI Profiling. **Table S6.** Patients with BOT Providing Sera for LMI Profiling. **Table S7.** Patients with PCL Providing Sera for LMI Profiling. **Table S8.** Patients with BRC Providing Sera for LMI Profiling. **Table S9.** Patients with BBT Providing Sera for LMI Profiling. **Table S10.** Patients with UCC Providing Sera for LMI Profiling. **Table S11.** Patients with EMC Providing Sera for LMI Profiling. (DOCX 145 kb)

## Abbreviations

BBT: Benign breast tumor
BOT: Benign ovarian tumor
BRC: Breast cancer
BUT: Benign uterine tumor
CA-125: Cancer antigen 125
CRC: Colorectal cancer
DS: Discriminant score
EMC: Endometrial cancer
FA: Formic acid
GC: Gastric cancer
HMDB: Human Metabolome Database
LC-MS: Liquid chromatography - mass spectrometry
LMI: Low-mass-ion
LOME: Low-mass-ion discriminant equation
LPC: Lysophosphatidylcholine
*m/z*: Mass-to-charge ratio
MALDI-TOF: Matrix-assisted laser desorption/ionization-time of flight
MS/MS: Tandem mass spectrometry
NPV: Negative predictive value
OVC: Ovarian cancer
PCA-DA: Principal component analysis - based discriminant analysis
PCL: Precancerous cervical lesion
PPV: Positive predictive value
TFA: Trifluoroacetic acid
UCC: Uterine cervical cancer
