# Supplementary material for: Low-mass-ion discriminant equation (LOME) for ovarian cancer screening
Source: BioData Min. 2016 Oct 12;9:32. doi: 10.1186/s13040-016-0111-7 (PMC5059959; doi:10.1186/s13040-016-0111-7)
Supplement: Additional file 1: — More detailed information for individual samples. Table S1.Healthy Control Individuals Providing Sera for LMI Profiling. Table S2. Patients with OVC Providing Sera for LMI Profiling. Table S3. Patients with CRC Providing Sera for LMI Profiling. Table S4. Patients with GC Providing Sera for LMI Profiling. Table S5. Patients with BUT Providing Sera for LMI Profiling. Table S6. Patients with BOT Providing Sera for LMI Profiling. Table S7. Patients with PCL Providing Sera for LMI Profiling. Table S8. Patients with BRC Providing Sera for LMI Profiling. Table S9. Patients with BBT Providing Sera for LMI Profiling. Table S10. Patients with UCC Providing Sera for LMI Profiling. Table S11. Patients with EMC Providing Sera for LMI Profiling. (DOCX 145 kb) [file 13040_2016_111_MOESM1_ESM.docx]

**Additional file 1: Table S1.** Healthy Control Individuals Providing Sera for LMI Profiling

| Set A_1_ | | | | | | | | | | | |
| --- | --- | --- | --- | --- | --- | --- | --- | --- | --- | --- | --- |
| Sample  identifier | Age  year | Sample  identifier | Age  year | Sample  identifier | Age  year | Sample  identifier | Age  year | Sample  identifier | Age  year | Sample  identifier | Age  year |
| A001 | 37 | A017 | 50 | A033 | 47 | A048 | 86 | A063 | 43 | A078 | 48 |
| A002 | 55 | A018 | 56 | A034 | 46 | A049 | 41 | A064 | 57 | A079 | 59 |
| A003 | 54 | A019 | 54 | A035 | 63 | A050 | 62 | A065 | 60 | A080 | 64 |
| A004 | 49 | A020 | 57 | A036 | 60 | A051 | 47 | A066 | 60 | A081 | 46 |
| A005 | 47 | A021 | 69 | A037 | 49 | A052 | 35 | A067 | 45 | A082 | 61 |
| A006 | 46 | A022 | 59 | A038 | 61 | A053 | 55 | A068 | 48 | A083 | 28 |
| A007 | 63 | A023 | 54 | A039 | 41 | A054 | 36 | A069 | 64 | A084 | 40 |
| A008 | 60 | A024 | 49 | A040 | 54 | A055 | 53 | A070 | 63 | A085 | 31 |
| A009 | 49 | A025 | 52 | A041 | 47 | A056 | 48 | A071 | 38 | A086 | 53 |
| A010 | 61 | A026 | 52 | A042 | 45 | A057 | 56 | A072 | 50 | A087 | 30 |
| A011 | 69 | A027 | 55 | A043 | 52 | A058 | 44 | A073 | 52 | A088 | 36 |
| A012 | 70 | A028 | 70 | A044 | 43 | A059 | 46 | A074 | 33 | A089 | 32 |
| A013 | 50 | A029 | 37 | A045 | 56 | A060 | 54 | A075 | 57 | A090 | 52 |
| A014 | 37 | A030 | 55 | A046 | 57 | A061 | 47 | A076 | 59 | A091 | 49 |
| A015 | 38 | A031 | 54 | A047 | 40 | A062 | 35 | A077 | 66 | A092 | 34 |
| A016 | 54 | A032 | 49 |  |  |  |  |  |  |  |  |
| Set A_2_ | | | | | | | | | | | |
| Sample  identifier | Age  year | Sample  identifier | Age  year | Sample  identifier | Age  year | Sample  identifier | Age  year | Sample  identifier | Age  year | Sample  identifier | Age  year |
| A093 | 57 | A109 | 60 | A125 | 49 | A140 | 38 | A155 | 56 | A170 | 49 |
| A094 | 69 | A110 | 49 | A126 | 52 | A141 | 50 | A156 | 51 | A171 | 55 |
| A095 | 59 | A111 | 61 | A127 | 52 | A142 | 52 | A157 | 51 | A172 | 32 |
| A096 | 54 | A112 | 69 | A128 | 55 | A143 | 58 | A158 | 48 | A173 | 52 |
| A097 | 49 | A113 | 70 | A129 | 70 | A144 | 44 | A159 | 32 | A174 | 32 |
| A098 | 52 | A114 | 50 | A130 | 50 | A145 | 42 | A160 | 57 | A175 | 46 |
| A099 | 52 | A115 | 37 | A131 | 59 | A146 | 61 | A161 | 63 | A176 | 42 |
| A100 | 55 | A116 | 38 | A132 | 55 | A147 | 36 | A162 | 50 | A177 | 68 |
| A101 | 70 | A117 | 54 | A133 | 35 | A148 | 61 | A163 | 55 | A178 | 57 |
| A102 | 37 | A118 | 50 | A134 | 50 | A149 | 31 | A164 | 56 | A179 | 43 |
| A103 | 55 | A119 | 56 | A135 | 43 | A150 | 55 | A165 | 52 | A180 | 43 |
| A104 | 54 | A120 | 54 | A136 | 43 | A151 | 46 | A166 | 43 | A181 | 43 |
| A105 | 49 | A121 | 57 | A137 | 44 | A152 | 55 | A167 | 38 | A182 | 68 |
| A106 | 47 | A122 | 69 | A138 | 49 | A153 | 64 | A168 | 67 | A183 | 36 |
| A107 | 46 | A123 | 59 | A139 | 39 | A154 | 33 | A169 | 42 | A184 | 54 |
| A108 | 63 | A124 | 54 |  |  |  |  |  |  |  |  |
| Set B | | | | | | | | | | | |
| Sample  identifier | Age  year | Sample  identifier | Age  year | Sample  identifier | Age  year | Sample  identifier | Age  year | Sample  identifier | Age  year | Sample  identifier | Age  year |
| B001 | 69 | B017 | 55 | B033 | 38 | B048 | 52 | B063 | 45 | B078 | 63 |
| B002 | 70 | B018 | 70 | B034 | 54 | B049 | 62 | B064 | 39 | B079 | 41 |
| B003 | 50 | B019 | 37 | B035 | 50 | B050 | 33 | B065 | 42 | B080 | 49 |
| B004 | 37 | B020 | 55 | B036 | 56 | B051 | 46 | B066 | 51 | B081 | 62 |
| B005 | 38 | B021 | 54 | B037 | 54 | B052 | 57 | B067 | 36 | B082 | 28 |
| B006 | 54 | B022 | 49 | B038 | 47 | B053 | 38 | B068 | 30 | B083 | 51 |
| B007 | 50 | B023 | 47 | B039 | 40 | B054 | 47 | B069 | 43 | B084 | 48 |
| B008 | 56 | B024 | 46 | B040 | 35 | B055 | 43 | B070 | 46 | B085 | 52 |
| B009 | 54 | B025 | 63 | B041 | 57 | B056 | 51 | B071 | 65 | B086 | 50 |
| B010 | 57 | B026 | 60 | B042 | 48 | B057 | 30 | B072 | 38 | B087 | 43 |
| B011 | 69 | B027 | 49 | B043 | 59 | B058 | 41 | B073 | 33 | B088 | 36 |
| B012 | 59 | B028 | 61 | B044 | 46 | B059 | 48 | B074 | 35 | B089 | 54 |
| B013 | 54 | B029 | 69 | B045 | 52 | B060 | 42 | B075 | 63 | B090 | 27 |
| B014 | 49 | B030 | 70 | B046 | 40 | B061 | 34 | B076 | 75 | B091 | 19 |
| B015 | 52 | B031 | 50 | B047 | 30 | B062 | 38 | B077 | 47 | B092 | 73 |
| B016 | 52 | B032 | 37 |  |  |  |  |  |  |  |  |

**Additional file 1: Table S2.** Patients with OVC Providing Sera for LMI Profiling

| Set A_1_ | | | |
| --- | --- | --- | --- |
| Sample  identifier | Age  year | Stage | Histology |
| A001 | 52 | IIA | Endometrioid adenocarcinoma |
| A002 | 63 | IV | Papillary serous adenocarcinoma |
| A003 | 55 | IA | Malignant Brenner tumour |
| A004 | 50 | IC | Clear cell carcinoma |
| A005 | 44 | IIIC | Papillary serous adenocarcinoma |
| A006 | 53 | IIIB | Serous carcinoma |
| A007 | 77 | IIIC | Transitional cell carcinoma |
| A008 | 57 | IIIC | Clear cell carcinoma |
| A009 | 51 | IIB | Serous carcinoma |
| A010 | 74 | IV | Serous carcinoma |
| A011 | 56 | IIIC | Serous carcinoma |
| A012 | 50 | IIIC | Serous carcinoma |
| A013 | 54 | IIIC | Serous carcinoma |
| A014 | 66 | IIIC | Serous carcinoma |
| A015 | 55 | IA | Clear cell carcinoma |
| A016 | 58 | IIIC | Serous carcinoma |
| A017 | 42 | IIIC | Serous carcinoma |
| A018 | 44 | IIA | Clear cell carcinoma |
| A019 | 54 | IIIC | Serous carcinoma |
| A020 | 34 | IA | Serous carcinoma |
| A021 | 41 | IA | Clear cell carcinoma |
| A022 | 61 | IIIC | Serous carcinoma |
| A023 | 65 | IIIC | Endometrioid carcinoma |
| A024 | 52 | IIIC | Serous carcinoma |
| A025 | 53 | IC | Serous carcinoma |
| A026 | 57 | IIIC | Metastatic papillary serous carcinoma |
| A027 | 57 | IV | Papillary serous carcinoma |
| A028 | 48 | IIIA2 | Clear cell carcinoma |
| A029 | 55 | IIIC | Papillary serous carcinoma |
| A030 | 52 | IV | Serous adenocarcinoma |
| Set A_2_ | | | |
| Sample  identifier | Age  year | Stage | Histology |
| A031 | 73 | IIIC | Serous adenocarcinoma |
| A032 | 47 | IIIC | Papillary serous adenocarcinoma |
| A033 | 58 | IIC | Serous adenocarcinoma |
| A034 | 57 | IB | Mixed cell adenocarcinoma |
| A035 | 68 | IA | Mucinous carcinoma |
| A036 | 67 | IIIC | Serous carcinoma |
| A037 | 74 | IIIC | Serous carcinoma |
| A038 | 67 | IV | Serous carcinoma |
| A039 | 57 | IIIC | Serous carcinoma |
| A040 | 57 | IIIC | Serous carcinoma |
| A041 | 61 | IIIA | Endometrioid carcinoma |
| A042 | 58 | IIA | Serous carcinoma |
| A043 | 55 | IV | Serous carcinoma |
| A044 | 51 | IIIC | Serous carcinoma |
| A045 | 57 | IV | Serous carcinoma |
| A046 | 52 | IC | Serous carcinoma |
| A047 | 27 | IA | Mucinous carcinoma |
| A048 | 52 | IA | Mucinous carcinoma |
| A049 | 41 | IIIC | Serous carcinoma |
| A050 | 46 | IIIC | Serous carcinoma |
| A051 | 69 | IIIC | Serous carcinoma |
| A052 | 57 | IIIC | Serous carcinoma |
| A053 | 62 | IIIC | Serous carcinoma |
| A054 | 73 | IIIB | Serous carcinoma |
| A055 | 71 | IIB | Serous carcinoma |
| A056 | 61 | IC1 | Serous carcinoma |
| A057 | 60 | IIIC | Papillary serous carcinoma |
| A058 | 51 | IIIC | Papillary serous carcinoma |
| A059 | 41 | IVB | Metastatic papillary serous carcinoma |
| A060 | 68 | IIIC | Serous carcinoma |
| Set B | | | |
| Sample  identifier | Age  year | Stage | Histology |
| B001 | 72 | IIIC | Serous adenocarcinoma |
| B002 | 54 | IIIC | Papillary serous adenocarcinoma |
| B003 | 40 | IC | Papillary serous adenocarcinoma |
| B004 | 74 | IIB | Transitional cell carcinoma |
| B005 | 47 | IV | Serous adenocarcinoma |
| B006 | 67 | IA | Mucinous carcinoma |
| B007 | 49 | IIIC | Serous carcinoma |
| B008 | 59 | IIIC | Serous carcinoma |
| B009 | 46 | IIA | Endometrioid carcinoma |
| B010 | 44 | IV | Mucinous carcinoma |
| B011 | 49 | IIIC | Serous carcinoma |
| B012 | 44 | IIIC | Serous carcinoma |
| B013 | 53 | IIIC | Serous carcinoma |
| B014 | 52 | IIIC | Serous carcinoma |
| B015 | 49 | IA | Endometrioid carcinoma |
| B016 | 64 | IIA | Serous carcinoma |
| B017 | 55 | IA | Serous carcinoma |
| B018 | 52 | IIIC | Endometrioid carcinoma |
| B019 | 43 | IC | Serous carcinoma |
| B020 | 49 | IIIC | Serous carcinoma |
| B021 | 37 | IIIC | Serous carcinoma |
| B022 | 54 | IIIA | Serous carcinoma |
| B023 | 48 | IIIC | Serous carcinoma |
| B024 | 76 | IIIB | Mucinous carcinoma |
| B025 | 75 | IIIA | Serous carcinoma |
| B026 | 45 | IC1 | Clear cell carcinoma |
| B027 | 51 | IIIC | Serous carcinoma |
| B028 | 56 | IIIC | Papillary serous carcinoma |
| B029 | 67 | IV | Serous carcinoma |

**Additional file 1: Table S3.** Patients with CRC Providing Sera for LMI Profiling

| Set A_1_ | | | | | | | |
| --- | --- | --- | --- | --- | --- | --- | --- |
| Sample  identifier | Age  year | Stage | Location | Sample  identifier | Age  year | Stage | Location |
| A001 | 48 | IIB | T-colon | A041 | 58 | IIIB | Colon |
| A002 | 50 | IIIA | Rectum | A042 | 56 | IIIB | Rectum |
| A003 | 73 | IIIA | Rectum | A043 | 67 | IIA | Colon |
| A004 | 70 | I | A-colon | A044 | 70 | IIA | Colon |
| A005 | 41 | I | HF-colon | A045 | 53 | IIA | Colon |
| A006 | 51 | IIIB | A-colon | A046 | 47 | I | Colon |
| A007 | 33 | IIIB | S-colon | A047 | 70 | IIA | Colon |
| A008 | 58 | I | RS-colon | A048 | 58 | IIA | Colon |
| A009 | 53 | IIIB | HF-colon | A049 | 71 | IIA | Colon |
| A010 | 58 | I | S-colon | A050 | 80 | IIIB | Colon |
| A011 | 82 | IIIB | Rectum | A051 | 77 | IIA | Colon |
| A012 | 44 | IIIB | S-colon | A052 | 73 | IVB | Colon |
| A013 | 70 | IIIB | S-colon | A053 | 72 | I | Rectum |
| A014 | 84 | IIB | S-colon | A054 | 50 | I | Rectum |
| A015 | 79 | 0 | S-colon | A055 | 71 | IIA | Rectum |
| A016 | 59 | IIIB | Rectum | A056 | 56 | IIIA | Colon |
| A017 | 48 | I | Rectum | A057 | 56 | IIIB | Colon |
| A018 | 52 | IIIC | S-colon | A058 | 56 | IIA | Colon |
| A019 | 53 | IIA | RS-colon | A059 | 55 | I | Rectum |
| A020 | 78 | I | Rectum | A060 | 75 | IIIB | Colon |
| A021 | 43 | IIA | RS-colon | A061 | 71 | I | Colon |
| A022 | 66 | IIA | S-colon | A062 | 69 | I | Rectum |
| A023 | 73 | IIA | HF-colon | A063 | 63 | IIIB | Colon |
| A024 | 68 | IV | S-colon | A064 | 74 | IVB | Rectum |
| A025 | 74 | IVA | Rectum | A065 | 71 | IIIA | Colon |
| A026 | 55 | IIIB | RS-colon | A066 | 66 | IIA | Colon |
| A027 | 53 | I | D-colon | A067 | 73 | IIB | Colon |
| A028 | 74 | I | Rectum | A068 | 78 | IIA | Colon |
| A029 | 80 | IIIB | S-colon | A069 | 63 | IIA | Rectum |
| A030 | 82 | IIIB | Rectum | A070 | 73 | I | Colon |
| A031 | 53 | IIIC | S-colon | A071 | 72 | I | Colon |
| A032 | 46 | IIIB | RS-colon | A072 | 55 | IIIB | Rectum |
| A033 | 81 | IIA | D-colon | A073 | 47 | I | Colon |
| A034 | 56 | IIIB | S-colon | A074 | 71 | IIA | Colon |
| A035 | 75 | I | A-colon | A075 | 43 | I | Rectum |
| A036 | 50 | IIA | S-colon | A076 | 55 | IIIA | Colon |
| A037 | 71 | IIIA | Colon | A077 | 55 | I | Rectum |
| A038 | 67 | IIIB | Colon | A078 | 74 | IIA | Rectum |
| A039 | 51 | IIIA | Rectum | A079 | 71 | I | Colon |
| A040 | 79 | I | Colon |  |  |  |  |
| Set A_2_ | | | | | | | |
| Sample  identifier | Age  year | Stage | Location | Sample  identifier | Age  year | Stage | Location |
| A080 | 71 | I | RS-colon | A120 | 41 | I | Colon |
| A081 | 45 | IIA | S-colon | A121 | 68 | I | Rectum |
| A082 | 36 | IIIB | S-colon | A122 | 76 | II | Colon |
| A083 | 73 | IIA | S-colon | A123 | 72 | I | Rectum |
| A084 | 86 | IIA | Rectum | A124 | 47 | I | Colon |
| A085 | 68 | IIIB | A-colon | A125 | 52 | IIA | Colon |
| A086 | 71 | I | S-colon | A126 | 40 | IIIC | Colon |
| A087 | 84 | IIIB | A-colon | A127 | 62 | IIIC | Colon |
| A088 | 69 | IIA | Rectum | A128 | 72 | IIIA | Rectum |
| A089 | 39 | IIA | Rectum | A129 | 50 | IIIB | Colon |
| A090 | 42 | IIIB | A-colon | A130 | 68 | IIA | Colon |
| A091 | 49 | IIIA | A-colon | A131 | 62 | IIIA | Rectum |
| A092 | 47 | IIIC | S-colon | A132 | 75 | IIIA | Colon |
| A093 | 78 | IIIB | RS-colon | A133 | 56 | I | Rectum |
| A094 | 65 | I | Rectum | A134 | 52 | IIA | Rectum |
| A095 | 54 | I | S-colon | A135 | 65 | I | Rectum |
| A096 | 59 | I | S-colon | A136 | 74 | IIIB | Rectum |
| A097 | 78 | IIA | S-colon | A137 | 81 | II | Colon |
| A098 | 72 | IIIB | Rectum | A138 | 70 | IIIC | Colon |
| A099 | 54 | IIIB | S-colon | A139 | 76 | IIA | Colon |
| A100 | 73 | I | A-colon | A140 | 59 | IVA | Colon |
| A101 | 54 | IVA | A-colon | A141 | 70 | IIIB | Rectum |
| A102 | 73 | IIIB | T-colon | A142 | 65 | III | Colon |
| A103 | 69 | I | A-colon | A143 | 56 | IIIC | Colon |
| A104 | 62 | IIIB | A-colon | A144 | 63 | II | Colon |
| A105 | 79 | IIA | RS-colon | A145 | 51 | I | Rectum |
| A106 | 57 | IIIC | A-colon | A146 | 30 | II | Rectum |
| A107 | 66 | IIA | S-colon | A147 | 65 | I | Colon |
| A108 | 65 | IIIB | Rectum | A148 | 46 | II | Rectum |
| A109 | 48 | IIIC | RS-colon | A149 | 56 | I | Colon |
| A110 | 63 | I | Rectum | A150 | 62 | IIIC | Colon |
| A111 | 55 | IIIB | RS-colon | A151 | 78 | I | Colon |
| A112 | 60 | I | S-colon | A152 | 49 | IIA | Colon |
| A113 | 46 | IIIC | Rectum | A153 | 55 | IIC | Colon |
| A114 | 38 | I | S-colon | A154 | 78 | IIIB | Rectum |
| A115 | 77 | II | Rectum | A155 | 85 | IIA | Colon |
| A116 | 82 | II | Colon | A156 | 73 | I | Rectum |
| A117 | 76 | IVA | Colon | A157 | 76 | IIA | Rectum |
| A118 | 74 | IIC | Colon | A158 | 63 | I | Colon |
| A119 | 72 | I | Colon |  |  |  |  |
| Set B | | | | | | | |
| Sample  identifier | Age  year | Stage | Location | Sample  identifier | Age  year | Stage | Location |
| B001 | 73 | IIIB | S-colon | B041 | 71 | I | Colon |
| B002 | 45 | IIIC | A-colon | B042 | 67 | IIA | Rectum |
| B003 | 52 | I | S-colon | B043 | 52 | I | Rectum |
| B004 | 58 | IIIB | S-colon | B044 | 72 | IIIB | Colon |
| B005 | 53 | I | A-colon | B045 | 74 | IIIB | Colon |
| B006 | 70 | I | Rectum | B046 | 74 | IIA | Colon |
| B007 | 66 | IIIA | S-colon | B047 | 75 | IIB | Colon |
| B008 | 75 | IIIB | S-colon | B048 | 69 | IIIB | Colon |
| B009 | 54 | I | Rectum | B049 | 75 | I | Colon |
| B010 | 70 | IIIB | S-colon | B050 | 58 | I | Colon |
| B011 | 72 | IIIB | A-colon | B051 | 40 | IIA | Colon |
| B012 | 73 | IIA |  | B052 | 66 | I | Colon |
| B013 | 63 | I | S-colon | B053 | 59 | II | Colon |
| B014 | 49 | IIA | S-colon | B054 | 51 | IIA | Rectum |
| B015 | 74 | IIB | HF-colon | B055 | 59 | IIB | Colon |
| B016 | 76 | IIIB | HF-colon | B056 | 72 | IIC | Colon |
| B017 | 58 | I | Rectum | B057 | 74 | I | Colon |
| B018 | 64 | IIIC | RS-colon | B058 | 71 | IIA | Rectum |
| B019 | 58 | IIA | S-colon | B059 | 76 | IIIB | Colon |
| B020 | 65 | 0 | Rectum | B060 | 54 | I | Rectum |
| B021 | 66 | IIB | T-colon | B061 | 43 | II | Colon |
| B022 | 62 | IIA | T-colon | B062 | 57 | I | Rectum |
| B023 | 48 | IIIB | S-colon | B063 | 65 | I | Rectum |
| B024 | 53 | IVA | Rectum | B064 | 70 | IIIB | Rectum |
| B025 | 79 | IVB | A-colon | B065 | 76 | IVB | Colon |
| B026 | 54 | IIIB | A-colon | B066 | 68 | I | Colon |
| B027 | 65 | IIIB | RS-colon | B067 | 72 | IIA | Rectum |
| B028 | 87 | IIA | RS-colon | B068 | 68 | IIIB | Rectum |
| B029 | 82 | IIA | Rectum | B069 | 72 | IIIB | Rectum |
| B030 | 59 | I | RS-colon | B070 | 55 | I | Rectum |
| B031 | 73 | IIIA | RS-colon | B071 | 44 | I | Colon |
| B032 | 64 | IIIB | S-colon | B072 | 73 | I | Colon |
| B033 | 53 | IIIB | S-colon | B073 | 70 | IIIC | Rectum |
| B034 | 65 | I | Rectum | B074 | 69 | IIIC | Colon |
| B035 | 78 | I | Rectum | B075 | 43 | III | Rectum |
| B036 | 58 | IVB | Colon | B076 | 63 | IIIC | Rectum |
| B037 | 54 | IIA | Colon | B077 | 49 | IIIA | Colon |
| B038 | 67 | IIA | Colon | B078 | 72 | IIIC | Rectum |
| B039 | 74 | II | Colon | B079 | 56 | IIIB | Rectum |
| B040 | 69 | IIA | Colon |  |  |  |  |

**Additional file 1: Table S4.** Patients with GC Providing Sera for LMI Profiling

| Set A_1_ | | | | | | | | | | | |
| --- | --- | --- | --- | --- | --- | --- | --- | --- | --- | --- | --- |
| Sample  identifier | Age  year | Stage | Sample  identifier | Age  year | Stage | Sample  identifier | Age  year | Stage | Sample  identifier | Age  year | Stage |
| A001 | 62 | IA | A013 | 70 | IA | A025 | 55 | IA | A037 | 82 | IIA |
| A002 | 67 | IA | A014 | 72 | IA | A026 | 54 | IA | A038 | 52 | IV |
| A003 | 64 | IB | A015 | 64 | IA | A027 | 56 | IA | A039 | 35 | IA |
| A004 | 41 | IB | A016 | 36 | IA | A028 | 72 | IIIA | A040 | 44 | IA |
| A005 | 81 | IIIB | A017 | 73 | IA | A029 | 76 | IA | A041 | 40 | IA |
| A006 | 79 | IA | A018 | 59 | IB | A030 | 82 | IIA | A042 | 66 | IA |
| A007 | 42 | IA | A019 | 81 | IV | A031 | 49 | IIIA | A043 | 38 | IV |
| A008 | 42 | IB | A020 | 86 | IV | A032 | 35 | IIB | A044 | 74 | IA |
| A009 | 54 | IA | A021 | 45 | IV | A033 | 62 | IA | A045 | 77 | IA |
| A010 | 45 | IIIC | A022 | 60 | IIB | A034 | 54 | IA | A046 | 55 | IIIB |
| A011 | 56 | IA | A023 | 81 | IA | A035 | 67 | IA | A047 | 56 | 0 |
| A012 | 61 | IA | A024 | 70 | IA | A036 | 64 | IA |  |  |  |
| Set A_2_ | | | | | | | | | | | |
| Sample  identifier | Age  year | Stage | Sample  identifier | Age  year | Stage | Sample  identifier | Age  year | Stage | Sample  identifier | Age  year | Stage |
| A048 | 62 | IA | A060 | 67 | IA | A072 | 53 | IA | A083 | 60 | IB |
| A049 | 65 | IA | A061 | 58 | IIIA | A073 | 70 | IA | A084 | 46 | IA |
| A050 | 54 | IA | A062 | 75 | IA | A074 | 57 | IB | A085 | 42 | IA |
| A051 | 58 | IIB | A063 | 68 | IA | A075 | 50 | IA | A086 | 77 | IIA |
| A052 | 40 | IA | A064 | 60 | IIIB | A076 | 40 | IV | A087 | 69 | IIIB |
| A053 | 74 | IV | A065 | 60 | IA | A077 | 71 | IA | A088 | 79 | IB |
| A054 | 77 | IV | A066 | 63 | IA | A078 | 48 | IIB | A089 | 52 | IIB |
| A055 | 28 | IA | A067 | 48 | IA | A079 | 42 | IA | A090 | 50 | IV |
| A056 | 70 | IIIB | A068 | 64 | IB | A080 | 46 | IB | A091 | 62 | IA |
| A057 | 38 | IV | A069 | 75 | IIIA | A081 | 59 | IA | A092 | 63 | IA |
| A058 | 72 | IA | A070 | 80 | IA | A082 | 69 | IA | A093 | 41 | IA |
| A059 | 62 | IA | A071 | 72 | IA |  |  |  |  |  |  |
| Set B | | | | | | | | | | | |
| Sample  identifier | Age  year | Stage | Sample  identifier | Age  year | Stage | Sample  identifier | Age  year | Stage | Sample  identifier | Age  year | Stage |
| B001 | 66 | IV | B013 | 39 | IA | B025 | 57 | IA | B036 | 46 | IA |
| B002 | 75 | IB | B014 | 76 | IIA | B026 | 39 | IA | B037 | 77 | IA |
| B003 | 65 | IA | B015 | 71 | IV | B027 | 63 | IV | B038 | 76 | IA |
| B004 | 71 | IA | B016 | 56 | IA | B028 | 45 | IA | B039 | 51 | IA |
| B005 | 68 | IA | B017 | 78 | IA | B029 | 58 | IV | B040 | 35 | IA |
| B006 | 43 | IA | B018 | 37 | IA | B030 | 41 | IIIA | B041 | 49 | IA |
| B007 | 50 | IA | B019 | 57 | IA | B031 | 76 | IIB | B042 | 57 | IA |
| B008 | 59 | IA | B020 | 58 | IA | B032 | 74 | IIA | B043 | 81 | IIIA |
| B009 | 63 | IA | B021 | 42 | IA | B033 | 81 | IB | B044 | 36 | IIIB |
| B010 | 66 | IA | B022 | 56 | IIIB | B034 | 58 | IA | B045 | 33 | IV |
| B011 | 30 | IA | B023 | 50 | IA | B035 | 69 | IA | B046 | 63 | 0 |
| B012 | 47 | IA | B024 | 53 | IIIB |  |  |  |  |  |  |

**Additional file 1: Table S5.** Patients with BUT Providing Sera for LMI Profiling

| Set A_1_ | | | | | | | | | | | | | | | |
| --- | --- | --- | --- | --- | --- | --- | --- | --- | --- | --- | --- | --- | --- | --- | --- |
| Sample  identifier | | Age  year | | Pathology | Sample  identifier | | Age  year | | Pathology | Sample  identifier | | Age  year | | Pathology | |
| A001 | | 41 | | Leiomyoma | A010 | | 39 | | Leiomyoma | A019 | | 47 | | Leiomyoma | |
| A002 | | 52 | | Adenomyosis | A011 | | 48 | | Leiomyoma | A020 | | 44 | | Leiomyoma | |
| A003 | | 42 | | Adenomyosis | A012 | | 38 | | Leiomyoma | A021 | | 39 | | Leiomyoma | |
| A004 | | 32 | | Leiomyoma | A013 | | 57 | | Leiomyoma | A022 | | 38 | | Leiomyoma | |
| A005 | | 41 | | Leiomyoma | A014 | | 41 | | Leiomyoma | A023 | | 32 | | Leiomyoma | |
| A006 | | 45 | | Leiomyoma | A015 | | 34 | | Leiomyoma | A024 | | 45 | | Leiomyoma | |
| A007 | | 51 | | Leiomyoma | A016 | | 51 | | Leiomyoma | A025 | | 47 | | Leiomyoma | |
| A008 | | 42 | | Leiomyoma | A017 | | 42 | | Leiomyoma | A026 | | 35 | | Leiomyoma | |
| A009 | | 49 | | Leiomyoma | A018 | | 65 | | Leiomyoma | A027 | | 45 | | Leiomyoma | |
| Set A_2_ | | | | | | | | | | | | | | | |
| Sample  identifier | | Age  year | | Pathology | Sample  identifier | | Age  year | | Pathology | Sample  identifier | | Age  year | | Pathology | |
| A028 | | 70 | | Leiomyoma | A038 | | 44 | | Leiomyoma | A047 | | 48 | | Leiomyoma | |
| A029 | | 56 | | Leiomyoma | A039 | | 29 | | Leiomyoma | A048 | | 45 | | Leiomyoma | |
| A030 | | 50 | | Leiomyoma | A040 | | 25 | | Leiomyoma | A049 | | 56 | | Adenomyosis | |
| A031 | | 48 | | Adenomyosis | A041 | | 69 | | Leiomyoma | A050 | | 40 | | Leiomyoma | |
| A032 | | 51 | | Leiomyoma | A042 | | 48 | | Leiomyoma | A051 | | 44 | | Leiomyoma | |
| A033 | | 47 | | Leiomyoma | A043 | | 42 | | Leiomyoma | A052 | | 44 | | Leiomyoma | |
| A034 | | 50 | | Leiomyoma | A044 | | 51 | | Leiomyoma | A053 | | 47 | | Leiomyoma | |
| A035 | | 47 | | Leiomyoma | A045 | | 48 | | Leiomyoma | A054 | | 45 | | Leiomyoma | |
| A036 | | 67 | | Leiomyoma | A046 | | 51 | | Leiomyoma | A055 | | 40 | | Leiomyoma | |
| A037 | | 57 | | Leiomyoma |  | |  | |  |  | |  | |  | |
| Set B | | | | | | | | | | | | | | | |
| Sample  identifier | Age  year | | Pathology | | | Sample  identifier | | Age  year | Pathology | | Sample  identifier | | Age  year | | Pathology |
| B001 | 42 | | Leiomyoma | | | B011 | | 50 | Leiomyoma | | B020 | | 49 | | Leiomyoma |
| B002 | 36 | | Leiomyoma | | | B012 | | 50 | Leiomyoma | | B021 | | 39 | | Leiomyoma |
| B003 | 50 | | Leiomyoma | | | B013 | | 49 | Leiomyoma | | B022 | | 36 | | Leiomyoma |
| B004 | 48 | | Adenomyosis | | | B014 | | 39 | Leiomyoma | | B023 | | 48 | | Leiomyoma |
| B005 | 41 | | Adenomyosis | | | B015 | | 47 | Endometrial polyp | | B024 | | 59 | | Leiomyoma |
| B006 | 46 | | Chronic inflammation  with squamous metaplasia | | | B016 | | 32 | Leiomyoma | | B025 | | 44 | | Leiomyoma |
| B007 | 39 | | Leiomyoma | | | B017 | | 50 | Leiomyoma | | B026 | | 40 | | Leiomyoma |
| B008 | 46 | | Leiomyoma | | | B018 | | 56 | Leiomyoma | | B027 | | 47 | | Leiomyoma |
| B009 | 43 | | Leiomyoma | | | B019 | | 46 | Endometrial polyp | | B028 | | 45 | | Leiomyoma |
| B010 | 40 | | Leiomyoma | | |  | |  |  | |  | |  | |  |

**Additional file 1: Table S6.** Patients with BOT Providing Sera for LMI Profiling

| Set A_1_ | | | | | |
| --- | --- | --- | --- | --- | --- |
| Sample  identifier | Age  year | Pathology | Sample  identifier | Age  year | Pathology |
| A001 | 45 | Chronic salpingitis | A013 | 36 | Endometriotic cyst |
| A002 | 26 | Cystic teratoma | A014 | 23 | Mature cystic teratoma |
| A003 | 62 | Serous cystadenoma | A015 | 51 | Endometriotic cyst |
| A004 | 53 | Chronic salpingitis | A016 | 42 | Serous cystadenofibroma |
| A005 | 45 | Mucinous cystadenoma | A017 | 33 | Endometriotic cyst |
| A006 | 36 | Endometriosis | A018 | 31 | Endometriotic cyst |
| A007 | 38 | Acute serositis, Chronic endometritis, Cervicitis | A019 | 30 | Mature cystic teratoma |
| A008 | 29 | Endometriosis | A020 | 49 | Endometriotic cyst |
| A009 | 25 | Mature cystic teratoma | A021 | 56 | Serous cystadenoma |
| A010 | 54 | Fibrothecoma | A022 | 26 | Mature cystic teratoma |
| A011 | 57 | Mucinous cystadenoma | A023 | 29 | Endometriosis |
| A012 | 41 | Serous cystadenoma |  |  |  |
| Set A_2_ | | | | | |
| Sample  identifier | Age  year | Pathology | Sample  identifier | Age  year | Pathology |
| A024 | 45 | Mucinous cystadenoma | A036 | 45 | Endometriosis |
| A025 | 73 | Solitary fibrous tumor | A037 | 42 | Endometriosis |
| A026 | 43 | Endometriosis | A038 | 36 | Serous cystadenoma |
| A027 | 42 | Endometriosis | A039 | 43 | Serous cystadenoma |
| A028 | 52 | Pseudomyxoma peritonei, Small serous cystadenoma | A040 | 34 | Mucinous cystadenoma |
| A029 | 62 | Serous cystadenoma | A041 | 54 | Mucinous cystadenoma |
| A030 | 55 | Serous cystadenoma | A042 | 30 | Serous cystadenofibroma |
| A031 | 36 | Endometriotic cyst | A043 | 43 | Endometriosis |
| A032 | 29 | Cystic teratoma | A044 | 30 | Mature cystic teratoma |
| A033 | 24 | Endometriotic cyst | A045 | 72 | Simple mesothelial cyst |
| A034 | 36 | Endometriosis | A046 | 25 | Mature cystic teratoma |
| A035 | 24 | Multilocular peritoneal  inclusion cyst | A047 | 42 | Endometriosis |
| Set B | | | | | |
| Sample  identifier | Age  year | Pathology | Sample  identifier | Age  year | Pathology |
| B001 | 55 | Mature cystic teratoma | B013 | 32 | Endometriotic cyst |
| B002 | 83 | Serous cystadenoma | B014 | 60 | Serous cystadenoma |
| B003 | 41 | Endometriosis | B015 | 31 | Mature cystic teratoma |
| B004 | 39 | Endometriosis | B016 | 41 | Endometriosis |
| B005 | 71 | Abscess | B017 | 58 | Serous cystadenoma |
| B006 | 49 | Endometriosis | B018 | 31 | Endometriosis  with focal degenerative atypia |
| B007 | 48 | Paratubal cyst | B019 | 52 | Simple cyst with atrophic ovary |
| B008 | 49 | Adenofibroma, Paratubal cyst | B020 | 26 | Mature cystic teratoma |
| B009 | 34 | Endometriosis | B021 | 42 | Endometriotic cyst |
| B010 | 43 | Ovarian and adnexal tissue with endometriosis | B022 | 34 | Endometriosis |
| B011 | 45 | Endometriosis | B023 | 46 | Serous cystadenoma |
| B012 | 21 | Serous papillary adenofibroma | B024 | 20 | Cystic teratoma |

**Additional file 1: Table S7.** Patients with PCL Providing Sera for LMI Profiling

| Set A_1_ | | | | | |
| --- | --- | --- | --- | --- | --- |
| Sample  identifier | Age  year | Pathology | Sample  identifier | Age  year | Pathology |
| A001 | 40 | Adenocarcinoma in situ | A016 | 64 | Cervical intraepithelial neoplasia II |
| A002 | 44 | Cervical intraepithelial neoplasia III | A017 | 45 | Cervical intraepithelial neoplasia I |
| A003 | 50 | Carcinoma in situ | A018 | 40 | Cervical intraepithelial neoplasia II |
| A004 | 71 | Cervical intraepithelial neoplasia I | A019 | 48 | Cervical intraepithelial neoplasia I |
| A005 | 47 | Atypical squamous cells  of undetermined significance | A020 | 34 | Cervical intraepithelial neoplasia III |
| A006 | 29 | Cervical intraepithelial neoplasia I | A021 | 50 | Cervical intraepithelial neoplasia III |
| A007 | 46 | Cervical intraepithelial neoplasia I | A022 | 33 | Cervical intraepithelial neoplasia II |
| A008 | 54 | Atypical squamous cells,  cannot rule out high-grade  squamous intraepithelial lesion | A023 | 42 | Carcinoma in situ |
| A009 | 33 | Cervical intraepithelial neoplasia III | A024 | 70 | Cervical intraepithelial neoplasia III |
| A010 | 50 | Carcinoma in situ | A025 | 74 | Carcinoma in situ |
| A011 | 71 | Cervical intraepithelial neoplasia II | A026 | 31 | Cervical intraepithelial neoplasia II |
| A012 | 42 | Cervical intraepithelial neoplasia III | A027 | 30 | Cervical intraepithelial neoplasia III |
| A013 | 36 | Cervical intraepithelial neoplasia III | A028 | 39 | Cervical intraepithelial neoplasia II |
| A014 | 45 | Cervical intraepithelial neoplasia I | A029 | 48 | Cervical intraepithelial neoplasia III |
| A015 | 52 | Cervical intraepithelial neoplasia I | A030 | 41 | Carcinoma in situ |
| Set A_2_ | | | | | |
| Sample  identifier | Age  year | Pathology | Sample  identifier | Age  year | Pathology |
| A031 | 32 | Cervical intraepithelial neoplasia II | A046 | 42 | Cervical intraepithelial neoplasia I |
| A032 | 52 | Cervical intraepithelial neoplasia I-II | A047 | 31 | Cervical intraepithelial neoplasia I |
| A033 | 45 | Cervical intraepithelial neoplasia II | A048 | 60 | Carcinoma in situ |
| A034 | 51 | Cervical intraepithelial neoplasia III | A049 | 47 | Cervical intraepithelial neoplasia II |
| A035 | 41 | Cervical intraepithelial neoplasia II-III | A050 | 36 | Cervical intraepithelial neoplasia III |
| A036 | 32 | Cervical intraepithelial neoplasia III | A051 | 31 | Cervical intraepithelial neoplasia III |
| A037 | 36 | Cervical intraepithelial neoplasia II | A052 | 25 | Cervical intraepithelial neoplasia III |
| A038 | 29 | Cervical intraepithelial neoplasia III | A053 | 44 | Carcinoma in situ |
| A039 | 32 | Cervical intraepithelial neoplasia III | A054 | 37 | Cervical intraepithelial neoplasia III |
| A040 | 36 | Carcinoma in situ | A055 | 44 | Cervical intraepithelial neoplasia II |
| A041 | 33 | Cervical intraepithelial neoplasia III | A056 | 42 | Cervical intraepithelial neoplasia III |
| A042 | 34 | Cervical intraepithelial neoplasia I | A057 | 28 | Cervical intraepithelial neoplasia III |
| A043 | 40 | Carcinoma in situ | A058 | 49 | Cervical intraepithelial neoplasia I |
| A044 | 47 | Cervical intraepithelial neoplasia I | A059 | 51 | Carcinoma in situ |
| A045 | 46 | Cervical intraepithelial neoplasia I |  |  |  |
| Set B | | | | | |
| Sample  identifier | Age  year | Pathology | Sample  identifier | Age  year | Pathology |
| B001 | 34 | Cervical intraepithelial neoplasia III | B016 | 48 | Carcinoma in situ |
| B002 | 58 | Cervical intraepithelial neoplasia III | B017 | 22 | Carcinoma in situ |
| B003 | 26 | Cervical intraepithelial neoplasia III | B018 | 24 | Cervical intraepithelial neoplasia III |
| B004 | 41 | Carcinoma in situ | B019 | 37 | Cervical intraepithelial neoplasia III |
| B005 | 39 | Cervical intraepithelial neoplasia II | B020 | 58 | Cervical intraepithelial neoplasia III |
| B006 | 41 | Adenocarcinoma in situ | B021 | 39 | Cervical intraepithelial neoplasia II |
| B007 | 36 | Cervical intraepithelial neoplasia III | B022 | 48 | Cervical intraepithelial neoplasia II |
| B008 | 51 | Cervical intraepithelial neoplasia I | B023 | 38 | Cervical intraepithelial neoplasia I |
| B009 | 32 | Cervical intraepithelial neoplasia III | B024 | 27 | Cervical intraepithelial neoplasia III |
| B010 | 54 | Cervical intraepithelial neoplasia II-III | B025 | 40 | Cervical intraepithelial neoplasia I |
| B011 | 25 | Cervical intraepithelial neoplasia II | B026 | 30 | Carcinoma in situ |
| B012 | 79 | Cervical intraepithelial neoplasia II | B027 | 29 | Carcinoma in situ |
| B013 | 31 | Cervical intraepithelial neoplasia II | B028 | 49 | Cervical intraepithelial neoplasia II |
| B014 | 33 | Cervical intraepithelial neoplasia II | B029 | 53 | Cervical intraepithelial neoplasia I |
| B015 | 30 | Adenocarcinoma in situ |  |  |  |

**Additional file 1: Table S8.** Patients with BRC Providing Sera for LMI Profiling

| Set A_1_ | | | | | | | | | | | |
| --- | --- | --- | --- | --- | --- | --- | --- | --- | --- | --- | --- |
| Sample  identifier | Age  year | Stage | Sample  identifier | Age  year | Stage | Sample  identifier | Age  year | Stage | Sample  identifier | Age  year | Stage |
| A001 | 52 | IIA | A009 | 33 | IIA | A017 | 68 | IA | A025 | 38 | IIA |
| A002 | 52 | IIA | A010 | 44 | IA | A018 | 53 | IA | A026 | 40 | IIIA |
| A003 | 40 | IA | A011 | 29 | IA | A019 | 41 | 0 | A027 | 52 | IIA |
| A004 | 40 | IA | A012 | 45 | IB | A020 | 49 | IA | A028 | 50 | 0 |
| A005 | 51 | IIA | A013 | 42 | IA | A021 | 42 | IIA | A029 | 47 | 0 |
| A006 | 64 | IA | A014 | 41 | IA | A022 | 50 | IIA | A030 | 47 | IIIA |
| A007 | 57 | IA | A015 | 67 | IA | A023 | 46 | IIA | A031 | 42 | IIIA |
| A008 | 69 | 0 | A016 | 40 | IIB | A024 | 57 | IIA |  |  |  |
| Set A_2_ | | | | | | | | | | | |
| Sample  identifier | Age  year | Stage | Sample  identifier | Age  year | Stage | Sample  identifier | Age  year | Stage | Sample  identifier | Age  year | Stage |
| A032 | 58 | IIB | A040 | 55 | IA | A048 | 40 | IIIA | A056 | 54 | IIA |
| A033 | 51 | IIA | A041 | 41 | IIB | A049 | 67 | IA | A057 | 39 | IA |
| A034 | 46 | IIA | A042 | 52 | IA | A050 | 43 | IIB | A058 | 44 | IA |
| A035 | 41 | IIB | A043 | 57 | IIB | A051 | 50 | IA | A059 | 69 | IA |
| A036 | 45 | IA | A044 | 33 | 0 | A052 | 46 | IIIA | A060 | 30 | IA |
| A037 | 35 | IB | A045 | 40 | IA | A053 | 37 | IIIA | A061 | 38 | IIA |
| A038 | 41 | IIB | A046 | 42 | 0 | A054 | 69 | IIB | A062 | 41 | 0 |
| A039 | 56 | IA | A047 | 52 | IA | A055 | 49 | IIA |  |  |  |
| Set B | | | | | | | | | | | |
| Sample  identifier | Age  year | Stage | Sample  identifier | Age  year | Stage | Sample  identifier | Age  year | Stage | Sample  identifier | Age  year | Stage |
| B001 | 46 | IA | B009 | 57 | IA | B017 | 59 | IA | B025 | 47 | IIB |
| B002 | 50 | IA | B010 | 52 | IIA | B018 | 41 | IA | B026 | 64 | IIIC |
| B003 | 55 | IA | B011 | 46 | IA | B019 | 51 | IIA | B027 | 52 | 0 |
| B004 | 66 | IA | B012 | 57 | IA | B020 | 54 | IA | B028 | 31 | IIIA |
| B005 | 42 | IIA | B013 | 50 | IA | B021 | 46 | IIA | B029 | 37 | 0 |
| B006 | 45 | IA | B014 | 44 | IIA | B022 | 51 | IIA | B030 | 43 | 0 |
| B007 | 33 | IB | B015 | 46 | IIA | B023 | 51 | IIA | B031 | 42 | 0 |
| B008 | 51 | IIA | B016 | 42 | IA | B024 | 48 | IIB |  |  |  |

**Additional file 1: Table S9.** Patients with BBT Providing Sera for LMI Profiling

| Set A_1_ | | | | | | | | | | | |
| --- | --- | --- | --- | --- | --- | --- | --- | --- | --- | --- | --- |
| Sample  identifier | Age  year | Sample  identifier | Age  year | Sample  identifier | Age  year | Sample  identifier | Age  year | Sample  identifier | Age  year | Sample  identifier | Age  year |
| A001 | 42 | A005 | 55 | A009 | 42 | A013 | 40 | A017 | 48 | A020 | 46 |
| A002 | 62 | A006 | 56 | A010 | 42 | A014 | 55 | A018 | 21 | A021 | 49 |
| A003 | 22 | A007 | 62 | A011 | 46 | A015 | 54 | A019 | 41 | A022 | 48 |
| A004 | 47 | A008 | 19 | A012 | 39 | A016 | 52 |  |  |  |  |
| Set A_2_ | | | | | | | | | | | |
| Sample  identifier | Age  year | Sample  identifier | Age  year | Sample  identifier | Age  year | Sample  identifier | Age  year | Sample  identifier | Age  year | Sample  identifier | Age  year |
| A023 | 58 | A027 | 27 | A031 | 53 | A035 | 49 | A038 | 32 | A041 | 54 |
| A024 | 22 | A028 | 39 | A032 | 48 | A036 | 33 | A039 | 46 | A042 | 52 |
| A025 | 42 | A029 | 55 | A033 | 39 | A037 | 36 | A040 | 32 | A043 | 50 |
| A026 | 46 | A030 | 45 | A034 | 38 |  |  |  |  |  |  |
| Set B | | | | | | | | | | | |
| Sample  identifier | Age  year | Sample  identifier | Age  year | Sample  identifier | Age  year | Sample  identifier | Age  year | Sample  identifier | Age  year | Sample  identifier | Age  year |
| B001 | 49 | B005 | 53 | B009 | 41 | B013 | 41 | B017 | 52 | B020 | 46 |
| B002 | 53 | B006 | 59 | B010 | 38 | B014 | 46 | B018 | 61 | B021 | 59 |
| B003 | 42 | B007 | 46 | B011 | 54 | B015 | 54 | B019 | 40 | B022 | 60 |
| B004 | 46 | B008 | 38 | B012 | 47 | B016 | 42 |  |  |  |  |

**Additional file 1: Table S10.** Patients with UCC Providing Sera for LMI Profiling

| Set B | | | |
| --- | --- | --- | --- |
| Sample  identifier | Age  year | Stage | Pathology |
| B001 | 26 | IIB | Squamous cell carcinoma |
| B002 | 32 | IB2 | Squamous cell carcinoma |
| B003 | 71 | IIB | Squamous cell carcinoma |
| B004 | 34 | IA1 | Microinvasive squamous cell carcinoma |
| B005 | 50 | IB1 | Adenocarcinoma |
| B006 | 52 | IB | Squamous cell carcinoma |
| B007 | 55 | IB1 | Adenosquamous carcinoma |
| B008 | 45 | IIB |  |
| B009 | 40 | I | Endocervical adenocarcinoma |
| B010 | 43 | IB1 | Squamous cell carcinoma |
| B011 | 40 | IB1 | Squamous cell carcinoma |
| B012 | 43 | IB1 | Squamous cell carcinoma |
| B013 | 40 | IV | Squamous cell carcinoma |
| B014 | 45 | I | Squamous cell carcinoma |
| B015 | 46 | IB1 | Adenosquamous carcinoma |
| B016 | 64 | IB1 | Squamous cell carcinoma |
| B017 | 44 | IB1 | Mucinous adenocarcinoma |
| B018 | 43 | IIB | Squamous cell carcinoma |
| B019 | 44 | IA1 | Squamous cell carcinoma |
| B020 | 47 | IIB | Squamous cell carcinoma |
| B021 | 44 | IA1 | Microinvasive squamous cell carcinoma |
| B022 | 30 | IV | Adenosquamous cell carcinoma |
| B023 | 51 | IB1 | Squamous cell carcinoma |
| B024 | 50 | IB1 | Squamous cell carcinoma |
| B025 | 67 | IB1 | Squamous cell carcinoma |
| B026 | 70 | IIA | Squamous cell carcinoma |
| B027 | 74 | IIB | Squamous cell carcinoma |
| B028 | 74 | IIB | Squamous cell carcinoma |
| B029 | 73 | IB1 | Squamous cell carcinoma |
| B030 | 82 | IIB | Squamous cell carcinoma |
| B031 | 49 | IIIB | Squamous cell carcinoma |
| B032 | 72 | IIB | Microinvasive papillary squamous cell carcinoma |
| B033 | 32 | IA1 | Microinvasive squamous cell carcinoma |

**Additional file 1: Table S11.** Patients with EMC Providing Sera for LMI Profiling

| Set B | | | |
| --- | --- | --- | --- |
| Sample  identifier | Age  year | Stage | Pathology |
| B001 | 58 | IIIC | Endometrioid adenocarcinoma |
| B002 | 66 | IA | Endometrioid adenocarcinoma |
| B003 | 49 | IA | Endometrioid adenocarcinoma |
| B004 | 64 | IIIC2 | Metastatic carcinoma |
| B005 | 54 | IA | Endometrioid adenocarcinoma |
| B006 | 52 | IA | Endometrioid adenocarcinoma |
| B007 | 54 | IA | Endometrioid adenocarcinoma |
| B008 | 54 | IA | Endometrioid adenocarcinoma |
| B009 | 40 | IA | Endometrioid adenocarcinoma |
| B010 | 45 | IA | Endometrioid adenocarcinoma |
